# Eltrombopag treatment of patients with secondary immune thrombocytopenia: retrospective EHR analysis

**DOI:** 10.1007/s00277-021-04637-2

**Published:** 2021-09-10

**Authors:** Pallavi Patwardhan, Adrienne Landsteiner, Lincy S. Lal, Lincy Geevarghese, Lisa Le, Savita Nandal, Adam Cuker

**Affiliations:** 1 Novartis Pharmaceuticals Corporation, East Hanover, NJ USA; 2 Optum Health Economics and Outcomes Research, 11000 Optum Circle, Eden Prairie, MN 55344 USA; 3 University of Texas School of Public Health, Houston, TX USA; 4 Department of Medicine and Department of Pathology & Laboratory Medicine, Perelman School of Medicine, University of Pennsylvania, Philadelphia, PA USA

**Keywords:** Secondary immune thrombocytopenia, Eltrombopag, Platelet response, Electronic health records, Retrospective study

## Abstract

Immune thrombocytopenia (ITP) may occur in isolation (primary) or in association with a predisposing condition (secondary ITP [sITP]). Eltrombopag is a well-studied treatment for primary ITP, but evidence is scarce for sITP. We evaluated real-world use of eltrombopag for sITP using electronic health records. Eligible patients had diagnoses of ITP and a qualifying predisposing condition, and eltrombopag treatment. We described patient characteristics, treatment patterns, platelet counts, and thrombotic and bleeding events. We identified 242 eligible patients; the most common predisposing conditions were hepatitis C and systemic lupus erythematosus. Average duration of eltrombopag treatment was 6.1 months. Most (81.4%) patients achieved a platelet count ≥ 30,000/µL at a mean of 0.70 months, 70.2% reached ≥ 50,000/µL at a mean of 0.95 months, and 47.1% achieved a complete response of > 100,000/µL at a mean of 1.43 months after eltrombopag initiation. At eltrombopag discontinuation, 105 patients (43%) experienced a treatment-free period for a mean 3.3 months. Bleeding events occurred with similar frequency before and during eltrombopag treatment whereas thrombotic events were less frequent during eltrombopag treatment. Our results suggest similar rates of platelet response with eltrombopag in patients with sITP as compared with primary ITP. In addition, a treatment-free period is possible for a substantial minority of patients.

**Supplementary Information:**

The online version contains supplementary material available at 10.1007/s00277-021-04637-2.

## Introduction

Immune thrombocytopenia (ITP) is an acquired condition resulting from immune-mediated impairment of platelet production and/or peripheral platelet destruction. [1] ITP may occur in isolation (primary ITP), or in association with a predisposing condition (secondary ITP [sITP]). It has been estimated that approximately 20% of cases of ITP in adults are sITP. [2, 3] Conditions associated with sITP include broader autoimmune diseases such as systemic lupus erythematosus (SLE), [4] Evans syndrome, and antiphospholipid syndrome (APS); immune deficiencies such as common variable immune deficiency (CVID), [5] selective IgA deficiency, and autoimmune lymphoproliferative syndrome [6]; infection with hepatitis C, [7, 8] human immunodeficiency virus, [9] and *Helicobacter pylori* [10]; and certain lymphoproliferative disorders such as chronic lymphocytic leukemia (CLL) [11] and small lymphocytic lymphoma (SLL).

The thrombopoietin receptor agonist (TPO-RA), eltrombopag, is an option for second-line treatment of primary ITP. [12] Its use was approved by the United States Food and Drug Administration (US FDA) in 2008, based on clinical trials comparing eltrombopag with placebo among patients with primary ITP. However, patients with secondary ITP were excluded. [13–15].

Eltrombopag has been used successfully in sITP associated with CLL, SLE, Evans syndrome, HIV, and APS. [16] However, to date, the accumulated evidence represents a small number of patients, primarily case reports. In the current study, we evaluated the real-world use of eltrombopag among patients with ITP secondary to a variety of predisposing conditions using an electronic health records (EHR) dataset. We aimed to describe patients with sITP treated with eltrombopag, evaluate clinical outcomes including platelet counts and thrombotic or bleeding events, and characterize treatment patterns including duration of eltrombopag therapy and attainment of a treatment-free period.

## Methods

### Data source

This study was performed using EHR data obtained from the Optum Clinical Database, which aggregates clinical records from a network of more than 140,000 providers at more than 700 hospitals and 7000 clinics and contains data for more than 64 million unique US patients. A proprietary deterministic matching technology allows linkage among different sources to cover the full spectrum of health care. The data are structured in a relational database comprising tables constructed from different parts of EHRs, different electronic medical records systems (e.g., prescribed medications, diagnosis codes, health care encounters, and laboratory test results), and select components of physician notes.

### Patient selection criteria

Eligible patients were required to have a diagnosis of ITP as confirmed by ICD-9/10 code (Appendix Table A1); a qualifying predisposing condition (SLE, Evans syndrome, antiphospholipid syndrome, CVID, selective IgA deficiency, autoimmune lymphoproliferative syndrome, HIV, HCV, CLL, and SLL) as indicated by ICD-9/10 code (Appendix Table A2); and treatment with eltrombopag. In addition, evidence of clinical activity in the EHR database was required for at least 3 months prior to and 6 months after the identified sITP diagnosis. Patients were excluded for pregnancy, clinical trial enrollment, or eltrombopag use in the 3-month baseline period. We also excluded cases of ITP and *Helicobacter pylori* because, whereas evidence of an association is apparent in other parts of the world including Italy and Japan, there is not a clear association between these entities in the USA. [10].

### Study design

**Fig. 1:**
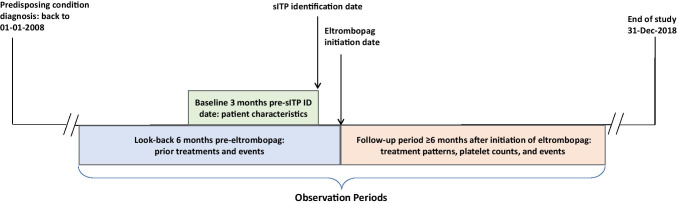
Study design and time periods designated for baseline and follow-up data collection. sITP, secondary immune thrombocytopenia. The sITP identification date is the first date on which an ITP diagnosis code was observed after the qualifying predisposing condition was diagnosed. The earliest ITP diagnosis may have occurred before the qualifying predisposing condition diagnosis, but inclusion criteria required both diagnoses present prior to the eltrombopag treatment period observed for this study

The entire period for this descriptive retrospective study encompassed 11 years between 01 January 2008 and 31 December 2018. For patients included in the study, observation started with diagnosis of a qualifying predisposing condition known to be associated with ITP. Following diagnosis of a qualifying condition, the first date on which an ITP diagnosis code was listed set the *sITP identification date*. All subjects were observed for a 3-month baseline period immediately before the sITP identification date for patient characteristics (Fig. 1).

The date on which eltrombopag treatment was initiated was set as the *eltrombopag initiation date*. Patients were observed for a 6-month look-back period prior to this date to identify previous treatment(s) and bleeding or thrombotic events and for at least 6 months after initiation of eltrombopag for observation of treatment patterns and outcomes. These time periods were chosen as optimal for the type of data needed. Treatment patterns were observed until disenrollment from the database, death, or end of study on 31 December 2018.

### Study variables

#### Patient characteristics

The following patient characteristics were collected in the baseline period: age, race/ethnicity, predisposing condition, health insurance type (commercial, Medicare, Medicaid, uninsured, other/unknown), and Quan-Charlson comorbidity score. [17] Length of time for which patients had continuous activity in the EHR database, reaching back to the start date of the study, was also recorded.

#### Treatment patterns

Pharmacy and medical orders were accessed to observe use of eltrombopag as monotherapy or in combination with other ITP treatments such as rituximab, romiplostim, splenectomy, corticosteroids, intravenous immune globulin (IVIG), anti-D, or fostamatinib. The eltrombopag treatment period began on the date of the first order for eltrombopag after the diagnosis of sITP. The observed eltrombopag treatment period ended by several criteria: death; discontinuation (90 days of no eltrombopag orders); start or addition of a new ITP therapy; end of the study period; or absence of EHR activity. As long as the patient remained alive and active in the EHR database, treatment patterns were observed until the end of the study period.

The use of systemic corticosteroids, IVIG, or anti-D was captured and described as *rescue medication* when it occurred after the start of the eltrombopag treatment period. The proportion of patients who initiated a second line of therapy (eltrombopag, rituximab, romiplostim, or splenectomy) following the observed eltrombopag treatment period was calculated. The time (months) between the end of the eltrombopag treatment period and the start of a subsequent treatment (eltrombopag restarted > 90 days after the original eltrombopag treatment period or initiation of another second-line treatment), as well as the length of each, were captured. The span of time after eltrombopag treatment ended, during which no rescue medication nor other ITP regimen was ordered, was reported as “treatment-free.”

### Outcomes

#### Platelet counts

All available platelet counts for the baseline period and follow-up period were captured. Mean platelet counts were calculated for a period within 14 days prior to initiation of eltrombopag therapy. From the initiation date of eltrombopag, mean platelet counts were calculated for the entire eltrombopag treatment period, as well as for a 6-month span. In addition, the proportion of patients reaching prespecified platelet count response levels and the time to those responses were noted. A first-level response was defined as ≥ 30,000/µL; a second-level response as ≥ 50,000/µL; and a complete response as ≥ 100,000/µL.

#### Thromboembolic events

During the follow-up period, arterial and venous thromboembolic events (thrombotic stroke, transient ischemic attack, myocardial infarction, deep vein thrombosis, and pulmonary embolism), based on ICD codes (Appendix Table A3), were captured.

#### Bleeding events

Bleeding and hemorrhagic events were also identified by ICD codes (Appendix Table A4).

#### Statistical analysis

Analysis was primarily descriptive, reporting proportions and mean (standard deviation [SD]) and median (minimum, maximum) as appropriate for patient characteristics, treatment patterns, and platelet counts. Chi-square and Fisher’s exact tests were used to compare the incidence of bleeding events and thrombotic events prior to and after the initiation of eltrombopag treatment. Because the sample was small, we used the Wilcoxin signed rank test to compare platelet values before and after initiation of eltrombopag. A *p*-value < 0.05 was considered statistically significant. All analyses were performed using SAS v.9.0 (SAS Institute, Cary, NC).

## Results

### Study sample

**Fig. 2:**
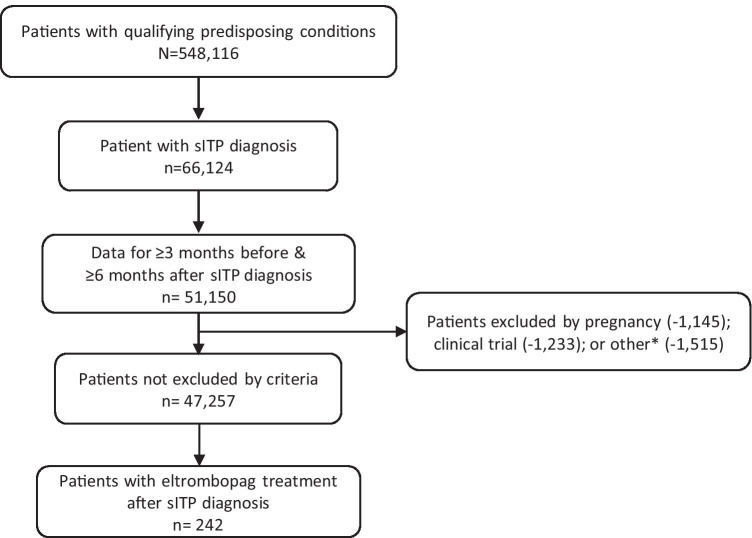
Sample selection and attrition process for identification of eligible patients. sITP, secondary immune thrombocytopenia. *Other exclusion criteria were multiple identification numbers, missing demographic data, or eltrombopag use prior to sITP diagnosis

We identified 51,150 patients with a qualifying predisposing condition, sITP diagnosis, and enrollment in the EHR for at least 3 months before and 6 months after the sITP diagnosis date. After exclusion criteria were applied, there were 47,257 patients, of whom 242 were prescribed eltrombopag and comprised our study population (Fig. 2).

The average age of patients was 52.5 years, and 50.8% were female (Table 1). Continuous clinical activity in the EHR database was evident for a mean (SD) 55.5 (32.3) months before and 48.3 (27.1) months after the sITP identification date. The most frequently observed predisposing conditions were HCV, SLE, CLL/SLL, APS, and Evans syndrome.

**Table 1:** Patient characteristics

	*N* = 242
Age (continuous), years	
Mean (SD)	52.5 (18.0)
Gender, female, *n* (%)	123 (50.8)
Insurance type, *n* (%)	
Commercial	70 (28.9)
Medicaid	43 (17.8)
Medicare	53 (21.9)
Commercial/Medicaid	8 (3.3)
Commercial/Medicare	19 (7.9)
Medicare/Medicaid	8 (3.3)
Commercial/Medicare/Medicaid	2 (0.8)
Invalid, missing, unknown, other	16 (6.6)
Uninsured	23 (9.5)
Race, *n* (%)	
Asian	3 (1.2)
Black or African American	36 (14.9)
White or Caucasian	186 (76.9)
Unknown/other	17 (7.0)
Ethnicity, *n* (%)	
Hispanic, Latino, or Spanish	24 (9.9)
Not Hispanic, Latino, or Spanish	210 (86.8)
Unknown/other	8 (3.3)
Charlson Comorbidity Index score	
Mean (SD)	2.31 (1.97)
Underlying qualifying conditions,* *n* (%)	
Hepatitis C	108 (44.6)
SLE	44 (18.2)
CLL or SLL	37 (15.3)
APS	26 (10.7)
Evans syndrome	19 (7.9)
CVID	12 (5.0)
HIV	12 (5.0)
Selective IgA deficiency	7 (2.9)
Autoimmune lymphoproliferative disorder	2 (0.8)

*ALPS*, autoimmune lymphoproliferative syndrome; *APS*, antiphospholipid syndrome; *CLL*, chronic lymphocytic leukemia; *CVID*, common variable immune deficiency; *HCV*, hepatitis C virus; *HIV*, human immunodeficiency virus; *SD*, standard deviation; *SLE*, systemic lupus erythematosus; *SLL*, small lymphocytic lymphoma

^*^Percentages do not add to 100% because patients may have had more than one condition

### Treatment patterns

Eltrombopag therapy began a mean (SD) of 1.1 (4.2) months following the sITP diagnosis date (Table 2). The majority (*n* = 202; 83.5%) of patients were treated with eltrombopag monotherapy; the remainder were treated with eltrombopag in combination with one or more additional ITP therapies. The average eltrombopag therapy duration was 6.1 months. The therapy ended for 146 (60.3%) patients due to discontinuation of eltrombopag, 45 (18.6%) patients due to a change in treatment, 47 (19.4%) patients due to end of activity in the EHR or end of the study period, and 4 (1.7%) patients due to death.

**Table 2:** Treatment pattern data

	*N* = 242
Time from sITP diagnosis to eltrombopag treatment, months, mean (SD)	1.1 (4.2)
Duration of eltrombopag treatment, months, mean (SD)	6.1 (6.9)
Patients who received eltrombopag monotherapy, *n* (%)	202 (83.5)
Patients who received eltrombopag in combination with additional ITP therapy, *n* (%)	40 (16.5)
Reason for the end of observed eltrombopag treatment period, *n* (%)	
Discontinuation	146 (60.3)
Add-on or switch	45 (18.6)
End of activity in EHR database or end of the study period	47 (19.4)
Death	4 (1.7)
Individuals who discontinued* eltrombopag treatment, and entered a treatment-free period** during available follow-up, *n* (%)	105 (43.4)
Observed length of treatment-free period, months, mean (SD)	3.3 (3.4)
Individuals with another line of therapy after eltrombopag, *n* (%)	84 (34.7)
Time from end of eltrombopag treatment to start of subsequent line, months, mean (SD)	5.7 (10.3)

*sITP*, secondary immune thrombocytopenia; *SD*, standard deviation; *EHR*, electronic health record

^*^Indicates the observed eltrombopag treatment period did not end (was not censored) because of death, end date of study, absence from database, or switch/add-on of another ITP therapy. The end of treatment period would only have been due to a discontinuation of the medication

^**^A “treatment-free period” indicates that the patient had completed eltrombopag treatment and was receiving neither another secondary ITP treatment, nor any rescue medications (systemic corticosteroids, IVIG, or anti-D)

Among the 146 patients who discontinued eltrombopag, 105 patients stopped all therapy and entered a treatment-free period, which was observed through the available follow-up period. The mean (SD) length of the treatment-free period was 3.3 (3.4) months. Among the 86 patients who discontinued eltrombopag and had at least 12 months of follow-up available after the sITP identification date, the mean (SD) treatment-free period was 9.9 (2.6) months.

### Platelet response

**Fig. 3:**
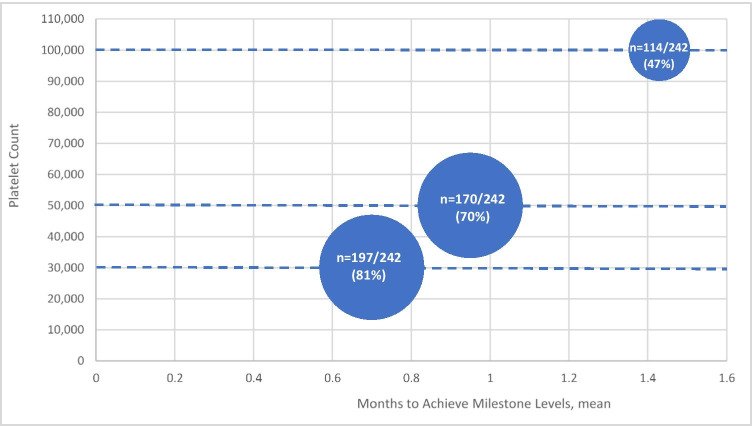
Time to threshold platelet count. Size of circles represent the proportion of the entire sample (81%, 70%, and 41%) which reached a given platelet count threshold (on the *y*-axis). The mean number of months to achieve that threshold is shown on the *x*-axis

Among the 242 patients, 166 had at least one platelet count in the 14 days prior to initiation of eltrombopag. For this set of 166 patients, the mean (SD; [median, minimum, maximum]) platelet count rose significantly from 45,000/uL (40,000/uL [33,000/uL, 3500/uL, 251,000/uL]) prior to starting eltrombopag to 76,000/uL (70,000/uL [52,000/uL, 3000 /uL, 375,000/uL) during eltrombopag therapy (*p* < 0.0001). The mean (SD) number of platelet counts within 14 days prior to eltrombopag initiation was 2.95 (2.78), and during eltrombopag treatment was 16.89 (16.24). Among the entire sample of 242 patients, 197 (81.4%) reached a first response level of ≥ 30,000/µL at a mean of 0.70 months; 170 (70.2%) reached a second response level of ≥ 50,000/µL at a mean of 0.95 months; and 114 patients (47.1%) achieved a complete response of > 100,000/µL at a mean of 1.43 months after initiation of eltrombopag (Fig. 3). Mean (SD) platelet counts at 1 month, 3 months, and 6 months following initiation of eltrombopag were 62,000/µL (56,000), 79,000/µL (78,000), and 83,000/µL (82,000), respectively.

### Thrombotic and bleeding events

Among the 242 patients, a total of 38 patients had ≥ 1 thrombotic event during the baseline period (Table 3). Deep vein thrombosis and pulmonary embolism were the most common. During the eltrombopag treatment period, 19 patients had thrombotic events, most commonly deep vein thrombosis. The mean (SD) number of events per patient was 0.16 (0.46) before treatment and 0.08 (0.16) after the start of eltrombopag (*p* = 0.027). Only for pulmonary embolism were the proportions of patients significantly different with fewer events during the eltrombopag treatment period (3.72% vs. 0.41%, *p* = 0.012). Among all patients, 32.2% had one or more bleeding events in the 6 months before, and 27.7% of patients had one or more bleeding events in the 6 months after the start of eltrombopag treatment (*p* = 0.628).

**Table 3:** Thrombotic and bleeding events during study period, *n* = 242

	6-month baseline period before eltrombopag	Eltrombopag treatment period*‡	*p*-values
Thrombotic events, mean (SD) per patient	0.16 (0.46)	0.08 (0.31)	0.027
Patients with specific events, *n* (%)			
Thrombotic stroke	6 (2.48)	4 (1.65)	0.528
Transient ischemic attack	4 (1.65)	2 (0.83)	0.416
Myocardial infarction	4 (1.65)	3 (1.24)	0.706
Deep vein thrombosis	15 (6.20)	9 (3.72)	0.222
Pulmonary embolism	9 (3.72)	1 (0.41)	0.012
Bleeding and hemorrhage events, *n* (%) of patients with ≥ 1 event	78 (32.2)	67 (27.7)	0.628

*SD*, standard deviation

^*^These are new events not occurring during the baseline period; an event was not counted in both time periods

^‡^The mean length of the observed eltrombopag treatment period was 6.1 months

## Discussion

Eltrombopag has been demonstrated to be effective and well-tolerated in the treatment of primary ITP in clinical trials and real-world studies. [14, 18, 19] Whether the efficacy and safety of eltrombopag in primary ITP is applicable to patients with sITP is uncertain. We conducted a real-world study of eltrombopag treatment among 242 patients with sITP using EHR data. To our knowledge, this is the largest study of a TPO-RA for the treatment of sITP. As with primary ITP, we found that eltrombopag in patients with sITP was associated with improvement in the platelet count and the potential for a subsequent treatment-free period.

The primary goal of treatment with eltrombopag in ITP is an improvement in platelet count and a reduced risk of bleeding events. In this study, the mean (SD) platelet count increased from a pre-treatment level of 45,000/uL (40,000/uL) to 76,000/uL (70,000/uL) during the treatment period (*p* < 0.0001). The majority of patients (70%) reached a second-level platelet response of ≥ 50,000/µL in a mean time of 1.0 month and 47% achieved a complete platelet response (> 100,000/µL) at mean of 1.4 months. By comparison, in a real-world sample of 87 Spanish patients with sITP, 35% of patients reached a complete response, but their starting median platelet count (14,000/ µL) was lower than in our study. [16] Although differences in patient population and outcome definitions limit the ability to compare our findings with clinical trials of eltrombopag in primary ITP, the response rates we observed do not appear to be inferior to those reported in such trials. For example, Bussel and colleagues reported achievement of a platelet count of 50,000/µL or higher among 50% of patients receiving eltrombopag within 0.5 months. [14] In the phase III RAISE trial, 79% of eltrombopag-treated patients achieved this platelet count threshold. [18].

Notable results were observed in this study regarding durability of response after discontinuation of eltrombopag treatment. After discontinuing eltrombopag, 43% of patients entered a treatment-free period for a mean of 3.3 months. Among those with at least 12 months of follow-up, the mean treatment-free period was 9.9 months. These findings align with observations in primary ITP, which show that approximately one-third of patients on eltrombopag are ultimately able to sustain a durable platelet response off all treatment. [20–22].

Bleeding events in the 6 months prior to treatment with eltrombopag were not significantly reduced in the 6 months after the start of treatment (32% vs. 28%, *p* = 0.628). This may be because the observation period was of insufficient length to detect differences in bleeding or because platelet counts, while lower in the baseline period, were generally in a hemostatic range. Whether treatment with TPO-RAs increases thrombotic risk in patients with ITP remains a matter of controversy. [23] Reassuringly, compared with the 6-month baseline period, the mean number of thromboembolic events per patient was lower in the 6 months after initiation of eltrombopag (0.16 vs. 0.08, *p* = 0.027).

### Limitations

As in all analyses using EHR data, certain limitations should be considered when interpreting our findings, including (1) the possibility of coding errors in the data, (2) a code for a disease does not guarantee accurate diagnosis, and (3) prescription orders do not confirm accurate or complete filling or administration of a medication. Some findings could only be identified by ICD codes, without additional descriptors; for example, bleeding events did not include data on type or grade of bleeding. The study design limits the ability to determine risk of developing thromboembolism or other events as associated with sITP with unadjusted p-values. Furthermore, although we had initially planned to conduct subgroup analyses based on predisposing condition, we were unable to meet this objective due to small sample sizes for the various conditions. Thus, while our results may apply to sITP patients with predisposing conditions that were well-represented in our cohort such as HCV, SLE, and CLL/SLL, they may not apply to predisposing conditions that were underrepresented in our study. Further research is needed to determine the effectiveness and safety of eltrombopag in patients with sITP and specific predisposing conditions. Guidelines state that most adults with ITP do not warrant treatment unless the platelet count is less than 30,000/uL.^12^ The mean pre-treatment platelet count (45,000/uL) in our cohort was above this threshold, suggesting that at least some eltrombopag-treated patients may not have required therapy. Finally, we did not capture information on treatment for predisposing conditions (e.g., antiviral therapy for HCV) and how such treatment may have affected the platelet count.

### Conclusions

Our results suggest that, among patients with sITP, eltrombopag shows similar effectiveness in improving platelet counts, compared with patients with primary ITP. In addition, as with primary ITP, a treatment-free period is possible for a substantial minority of patients. Prospective clinical studies specifically designed to evaluate eltrombopag for the treatment of sITP are needed.

## Supplementary Information

Below is the link to the electronic supplementary material.

Supplementary file1 (DOCX 14 KB)

Supplementary file2 (DOCX 19 KB)

Supplementary file3 (XLSX 24 KB)

Supplementary file4 (XLSX 42 KB)

## Data Availability

Use of the proprietary data obtained from the Optum Research Database requires strictest data security and privacy protocols and a restrictive license agreement. Thus, data used to generate the results presented are cannot be disclosed publicly.
