# Supplementary material for: Eltrombopag treatment of patients with secondary immune thrombocytopenia: retrospective EHR analysis
Source: Ann Hematol. 2021 Sep 10;101(1):11–9. doi: 10.1007/s00277-021-04637-2 (PMC8720735; doi:10.1007/s00277-021-04637-2)
Supplement: Supplementary file 1 — Supplementary file1 (DOCX 14 KB) [file 277_2021_4637_MOESM1_ESM.docx]

**Appendix Table A1.** *ICD Codes Identifying Eligible Patients by ITP Diagnosis*

| **Code** | **Code Type** | **Description** | **Condition** | **Comment** |
| --- | --- | --- | --- | --- |
| 287.3 | ICD-9-CX | primary thrombocytopenia | thrombocytopenia |  |
| D69 | ICD-10-CX | primary thrombocytopenia | thrombocytopenia |  |
| 287.3 | ICD-9-CX | primary thrombocytopenia | thrombocytopenia | does not have an ICD-10-CX |
| 287.31 | ICD-9-CX | Immune thrombocytopenic purpura | thrombocytopenia |  |
| D69.3 | ICD-9-CX | Immune thrombocytopenic purpura | thrombocytopenia |  |
| 287.32 | ICD-9-CX | Evans Syndrome | thrombocytopenia |  |
| D69.41 | ICD-10-CX | Evans Syndrome | thrombocytopenia |  |
| 287.39 | ICD-9-CX | Other Primary thrombocytopenia | thrombocytopenia |  |
| D69.49 | ICD-10-CX | Other Primary thrombocytopenia | thrombocytopenia |  |
| 287.4 | ICD-9-CX | Secondary thrombocytopenia | thrombocytopenia |  |
| D69.59 | ICD-10-CX | Secondary thrombocytopenia | thrombocytopenia |  |
| 287.49 | ICD-9-CX | Other secondary thrombocytopenia | thrombocytopenia |  |
| D59.59 | ICD-10-CX | Other secondary thrombocytopenia | thrombocytopenia |  |
| 287.5 | ICD-9-CX | Thrombocytopenia, unspecified | thrombocytopenia |  |
| D69.6 | ICD10 | Thrombocytopenia, unspecified | thrombocytopenia |  |
