# Supplementary material for: Eltrombopag treatment of patients with secondary immune thrombocytopenia: retrospective EHR analysis
Source: Ann Hematol. 2021 Sep 10;101(1):11–9. doi: 10.1007/s00277-021-04637-2 (PMC8720735; doi:10.1007/s00277-021-04637-2)
Supplement: Supplementary file 2 — Supplementary file2 (DOCX 19 KB) [file 277_2021_4637_MOESM2_ESM.docx]

**Appendix Table A2.** *ICD Codes Identifying Eligible Patients by Underlying Qualifying Disease*

| **Code** | **Code Type** | **Description** | **Condition** | **Comment** |
| --- | --- | --- | --- | --- |
| M320 | ICD-10 Dx | Drug-induced systemic lupus erythematosus | SLE |  |
| M3210 | ICD-10 Dx | Systemic lupus erythematosus, organ or system involvement unspecified | SLE |  |
| M3211 | ICD-10 Dx | Endocarditis in systemic lupus erythematosus | SLE |  |
| M3212 | ICD-10 Dx | Pericarditis in systemic lupus erythematosus | SLE |  |
| M3213 | ICD-10 Dx | Lung involvement in systemic lupus erythematosus | SLE |  |
| M3214 | ICD-10 Dx | Glomerular disease in systemic lupus erythematosus | SLE |  |
| M3215 | ICD-10 Dx | Tubulo-interstitial nephropathy in systemic lupus erythematosus | SLE |  |
| M3219 | ICD-10 Dx | Other organ or system involvement in systemic lupus erythematosus | SLE |  |
| M328 | ICD-10 Dx | Other forms of systemic lupus erythematosus | SLE |  |
| M329 | ICD-10 Dx | Systemic lupus erythematosus, unspecified | SLE |  |
| 710.0 | ICD-9 Dx | Systemic lupus erythematosus | SLE |  |
| D6941 | ICD-10 Dx | Evans syndrome | Evans Syndrome |  |
| 287.32 | ICD-9 Dx | Evans' syndrome | Evans Syndrome |  |
| D6861 | ICD-10 Dx | Antiphospholipid syndrome | Antiphospholipid Syndrome |  |
| 289.81 | ICD-9 Dx | Primary hypercoagulable state | Antiphospholipid Syndrome | Non-specific Code |
| 279.06 | ICD-9 Dx | Common variable immunodeficiency | Common Variable Immune Deficiency |  |
| D830 | ICD-10 Dx | Common variable immunodeficiency with predominant abnormalities of B-cell numbers and function | Common Variable Immune Deficiency |  |
| D831 | ICD-10 Dx | Common variable immunodeficiency with predominant immunoregulatory T-cell disorders | Common Variable Immune Deficiency |  |
| D832 | ICD-10 Dx | Common variable immunodeficiency with autoantibodies to B- or T-cells | Common Variable Immune Deficiency |  |
| D838 | ICD-10 Dx | Other common variable immunodeficiencies | Common Variable Immune Deficiency |  |
| D839 | ICD-10 Dx | Common variable immunodeficiency, unspecified | Common Variable Immune Deficiency |  |
| D802 | ICD-10 Dx | Selective deficiency of immunoglobulin A [IgA] | Selective IgA deficiency |  |
| 279.01 | ICD-9 Dx | Selective IgA immunodeficiency | Selective IgA deficiency |  |
| 042 | ICD-9 Dx | Human immunodeficiency virus [HIV] | HIV |  |
| 079.53 | ICD-9 Dx | Human immunodeficiency virus, type 2 (HIV 2), in conditions classified elsewhere and of unspecified site | HIV | Type 2 |
| V08 | ICD-9 Dx | Asymptomatic HIV infection status | HIV |  |
| B20 | ICD-10 Dx | HIV disease | HIV |  |
| B9735 | ICD-10 Dx | Human immunodeficiency virus, type 2 [HIV 2] as the cause of diseases classified elsewhere | HIV | Type 2 |
| Z21 | ICD-10 Dx | Asymptomatic HIV infection status | HIV |  |
| 070.41 | ICD-9 Dx | Acute hepatitis C with hepatic coma | Hepatitis C |  |
| 070.44 | ICD-9 Dx | Chronic hepatitis C with hepatic coma | Hepatitis C |  |
| 070.51 | ICD-9 Dx | Acute hepatitis C without mention of hepatic coma | Hepatitis C |  |
| 070.54 | ICD-9 Dx | Chronic hepatitis C without mention of hepatic coma | Hepatitis C |  |
| 070.70 | ICD-9 Dx | Unspecified viral hepatitis C without hepatic coma | Hepatitis C |  |
| 070.71 | ICD-9 Dx | Unspecified viral hepatitis C with hepatic coma | Hepatitis C |  |
| V02.62 | ICD-9 Dx | Hepatitis C carrier | Hepatitis C | Carrier |
| B1710 | ICD-10 Dx | Acute hepatitis C without hepatic coma | Hepatitis C |  |
| B1711 | ICD-10 Dx | Acute hepatitis C with hepatic coma | Hepatitis C |  |
| B182 | ICD-10 Dx | Chronic viral hepatitis C | Hepatitis C |  |
| B1920 | ICD-10 Dx | Unspecified viral hepatitis C without hepatic coma | Hepatitis C |  |
| B1921 | ICD-10 Dx | Unspecified viral hepatitis C with hepatic coma | Hepatitis C |  |
| Z2252 | ICD-10 Dx | Carrier of viral hepatitis C | Hepatitis C | Carrier |
| 204.10 | ICD-9 Dx | Chronic lymphoid leukemia, without mention of having achieved remission | CLL/SLL |  |
| 204.11 | ICD-9 Dx | Chronic lymphoid leukemia in remission | CLL/SLL |  |
| 204.12 | ICD-9 Dx | Chronic lymphoid leukemia, in relapse | CLL/SLL |  |
| C9110 | ICD-10 Dx | Chronic lymphocytic leukemia of B-cell type not having achieved remission | CLL/SLL |  |
| C9111 | ICD-10 Dx | Chronic lymphocytic leukemia of B-cell type in remission | CLL/SLL |  |
| C9112 | ICD-10 Dx | Chronic lymphocytic leukemia of B-cell type in relapse | CLL/SLL |  |
| 287.30 | ICD-9 Dx | Primary thrombocytopenia, unspecified | Primary Thrombocytopenia, Unspecified | No ICD-10 Code |
| D693 | ICD-10 Dx | Immune thrombocytopenic purpura | Immune Thrombocytopenic Purpura |  |
| 287.31 | ICD-9 Dx | Immune thrombocytopenic purpura | Immune Thrombocytopenic Purpura |  |
| D6949 | ICD-10 Dx | Other primary thrombocytopenia | Other Primary Thrombocytopenia |  |
| 287.39 | ICD-9 Dx | Other primary thrombocytopenia | Other Primary Thrombocytopenia |  |
| D6959 | ICD-10 Dx | Other secondary thrombocytopenia | Other Secondary Thrombocytopenia |  |
| 287.49 | ICD-9 Dx | Other secondary thrombocytopenia | Other Secondary Thrombocytopenia |  |
| D696 | ICD-10 Dx | Thrombocytopenia, unspecified | Thrombocytopenia, Unspecified |  |
| 287.5 | ICD-9 Dx | Unspecified thrombocytopenia | Thrombocytopenia, Unspecified |  |
